# Review of the Current Landscape of the Potential of Nanotechnology for Future Malaria Diagnosis, Treatment, and Vaccination Strategies

**DOI:** 10.3390/pharmaceutics13122189

**Published:** 2021-12-17

**Authors:** Arnau Guasch-Girbau, Xavier Fernàndez-Busquets

**Affiliations:** 1 Barcelona Institute for Global Health (ISGlobal), Hospital Clínic—University of Barcelona, Rosselló 149-153, 08036 Barcelona, Spain; arnauguasch2046@gmail.com; 2 Nanomalaria Group, Institute for Bioengineering of Catalonia (IBEC), The Barcelona Institute of Science and Technology, Baldiri Reixac 10-12, 08028 Barcelona, Spain; 3 Nanoscience and Nanotechnology Institute (IN2UB), University of Barcelona, Martí i Franquès 1, 08028 Barcelona, Spain

**Keywords:** antimalarial drugs, malaria diagnosis, malaria prophylaxis, malaria therapy, nanocarriers, nanomedicine, nanotechnology, *Plasmodium*, targeted drug delivery

## Abstract

Malaria eradication has for decades been on the global health agenda, but the causative agents of the disease, several species of the protist parasite *Plasmodium*, have evolved mechanisms to evade vaccine-induced immunity and to rapidly acquire resistance against all drugs entering clinical use. Because classical antimalarial approaches have consistently failed, new strategies must be explored. One of these is nanomedicine, the application of manipulation and fabrication technology in the range of molecular dimensions between 1 and 100 nm, to the development of new medical solutions. Here we review the current state of the art in malaria diagnosis, prevention, and therapy and how nanotechnology is already having an incipient impact in improving them. In the second half of this review, the next generation of antimalarial drugs currently in the clinical pipeline is presented, with a definition of these drugs’ target product profiles and an assessment of the potential role of nanotechnology in their development. Opinions extracted from interviews with experts in the fields of nanomedicine, clinical malaria, and the economic landscape of the disease are included to offer a wider scope of the current requirements to win the fight against malaria and of how nanoscience can contribute to achieve them.

## 1. Introduction

**Figure 1:**
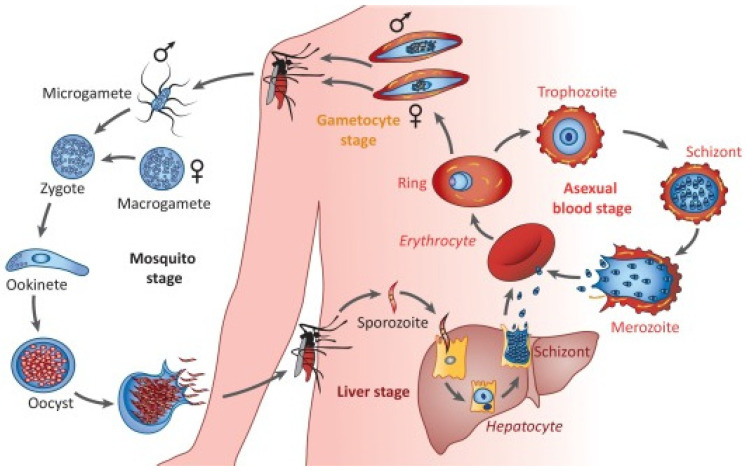
The life cycle of *Plasmodium falciparum*. Reproduced with permission from [4], Cell Press, 2018.

Malaria is an infectious disease caused by the parasite *Plasmodium* spp., which is transmitted by female *Anopheles* mosquitoes. Among the different *Plasmodium* species, five of them are known to infect humans: *P. falciparum*, *P. vivax*, *P. malariae*, *P. ovale*, and *P. knowlesi* [1], with the first two being the most prevalent and causing the majority of malaria cases [2]. *P. falciparum* is the most lethal species, causing severe clinical malaria, whereas *P. vivax* develops a dormant liver stage, the hypnozoite, which can trigger relapses after the primary infection [3]. The life cycle of *P. falciparum* [4,5,6] is shown in Figure 1.

Uncomplicated malaria patients present with a combination of fever, chills, sweats, headaches, nausea and vomiting, body aches, and general malaise [5]. Physical findings in uncomplicated malaria may include elevated temperatures, perspiration, weakness, enlarged spleen, mild jaundice, enlargement of the liver, and increased respiratory rate. Severe malaria occurs when infections are complicated by serious organ failures or abnormalities in the patient’s blood or metabolism. The manifestations of severe malaria include the following [5]: (i) cerebral malaria, with abnormal behavior, impairment of consciousness, seizures, coma, or other neurologic abnormalities; (ii) severe anemia due to destruction of RBCs; (iii) hemoglobinuria (hemoglobin in the urine) due to hemolysis; (iv) acute respiratory distress syndrome, an inflammatory reaction in the lungs that inhibits oxygen exchange, which may occur even after the parasite counts have decreased in response to treatment; (v) abnormalities in blood coagulation; (vi) low blood pressure caused by cardiovascular collapse; (vii) acute kidney injury; (viii) hyperparasitemia, in which more than 5% of RBCs are infected by malaria parasites; and (ix) metabolic acidosis (excessive acidity in the blood and tissue fluids), often in association with (x) hypoglycemia, which may also occur in pregnant women with uncomplicated malaria or after treatment with quinine. Severe malaria is a medical emergency and should be treated urgently and aggressively, e.g., with some of the drugs mentioned later in this review.

Malaria is classified by the World Health Organization (WHO) as a life-threatening disease that, despite being preventable and treatable, is still endemic in 87 countries, mainly in the tropical and subtropical regions [1]. The last WHO report on malaria estimated that in 2020, there were 241 million cases and 627,000 deaths worldwide, with the WHO African Region accounting for 95% of them [7]. Despite the decrease in disease burden and deaths experienced over the past years, the goal of malaria elimination is still far, mainly because of a lack of continued financing and the emergence of parasite resistance to the antimalarial drugs administered [1]. To achieve the global objective of malaria eradication, there is a need for research on the development of novel and affordable strategies for prevention and treatment, among which nanomedicine can play a pivotal role [8].

Nanomedicine can be defined as the application of nanotechnology to and the usage of nanomaterials for improvements in the health field. Traditionally, nanomedical tools have focused on noncommunicable diseases, with much effort on cancer [8,9]. However, recently, the application of nanomaterials to infectious and neglected diseases and to diseases of poverty, for example, malaria, has been extensively investigated and exploited [10,11,12].

There are different stages of the malaria parasite life cycle that can be targeted, potentially leading to the development of a new generation of antimalarial drugs [13]. The majority of efforts are concentrated on therapeutic applications, mainly on developing novel targeted drug delivery systems with the use of nanocarriers (NCs) [14,15]. These systems have the capacity to release the desired drug at a specific place with a high local dose, allowing rapid action against the pathogen without, or with very low, side effects [16]. This is possible because of the capacity of NCs to encapsulate several drug types and be functionalized with molecules to target the specific site of delivery. Moreover, NCs present good biological properties in terms of toxicity, half-life, and circulation time. Besides their therapeutic application, targeted drug delivery systems can also be used in prevention strategies, for example, by encapsulating the active molecule of a vaccine to trigger a stronger immune response [17]. In addition, nanotechnology can be applied to other malaria-related interventions, such as using green processes for the fabrication of nanoparticles (NPs) [18,19].

Although the research and development of novel drugs and strategies against malaria is a crucial issue for the elimination of this disease, there are other important factors that need to be assessed [20]: affordability, social and cultural factors, ethical considerations, surveillance systems, and resource allocation, to name a few, are relevant to eradication efforts [21,22].

## 2. Conventional Strategies against Malaria

A common trend among countries that have eliminated malaria recently is their heavy investment in control and prevention [23], the goal of which is to reduce disease prevalence [24] and limit the evolution of drug resistance by the parasite [25]. A robust monitoring and surveillance system is essential to progress towards control and elimination, allowing rapid case detection and appropriate response [26]. Active case detection is useful in elimination campaigns targeting hotspots and hot-pops, while passive surveillance is vital in any endemic country, with proper training of health workers where malaria cases are rare [23].

The integrated vector management (IVM) strategy is an inexpensive and efficient tool for vector control. IVM was first introduced by the WHO and uses a combination of interventions to attack different stages of the *Plasmodium* life cycle. Among all vector control tools, indoor residual spraying, long-lasting insecticidal nets, and insecticide-treated nets are the most widely implemented [27,28,29,30]. Combining these approaches with entomological surveillance [29], larva source management [30], insecticide rotation [25], and occupation-based vector control [23] has offered good results in different areas [25,27,29,31].

Of increasing interest as a chemoprevention tool is mass drug administration, defined as the administration of an antimalarial drug to an entire population, aiming at reducing disease prevalence [23,31]. In areas where transmission is high, the WHO recommends chemoprevention interventions as a prophylaxis tool for high-risk populations, including intermittent preventive treatment of infants (IPTi) and of pregnant women (IPTp) and seasonal malaria chemoprevention (SMC) for children under 5 years of age before and during the high transmission seasons [32], which have been proven effective, economical, and safe prophylactic strategies for the prevention of malaria in the targeted populations.

The most widely used tools for diagnosis are rapid diagnostic tests (RDTs). Although microscopy is used as well, there is limited access to health facilities having the necessary equipment and trained personnel. RDTs are the gold standard for malaria screening and diagnosis outside health centers, but they can detect only high parasite densities in people with symptomatic malaria [23,28,31]. The alternative tools are nucleic acid amplification tests (NAATs), which have advantages, such as high sensitivity and processivity and the capacity to identify drug-resistant strains, despite being more time consuming and expensive than RDTs [33].

Currently there are different natural and synthetic compounds available for the treatment of malaria, but their effectiveness has been decreasing, as *Plasmodium* has evolved resistance towards most of them. To reverse this trend, the WHO encourages using combination therapies, and some drugs have restricted usage only in severe situations when the combination therapy is not working [14,34]. The first natural product employed against malaria was quinine. Although it has been one of the most effective antimalarial treatments, resistance to it was reported during the 1980s, and since 2006, it has been used only for severe malaria cases when other treatments are not available [35]. In the same pharmacophore group of arylamine alcohols are lumefantrine, used for the treatment of uncomplicated *P. falciparum* malaria in combination with artemether [36], and mefloquine, which in combination with artesunate is used for the treatment of uncomplicated malaria [36,37]. Another important drug is chloroquine, used to treat all forms of malaria with few side effects, but to which resistance evolved during the 1950s [37]. Now it is used for the treatment of all uncomplicated malaria except for *P. falciparum* [36]. In the same quinoline chemical family are piperaquine and other drugs normally administered with artemisinin derivatives [34]. The WHO recommends the use of artemisinin and artemisinin-based combination therapy (ACT) for the treatment of malaria [36,38]. Artemisinin was first isolated during the early 1970s, showing efficacy even against multidrug-resistant forms of *P. falciparum* [37]. Among its several derivatives, the most common are artemether and artesunate, widely used in the treatment of all forms of uncomplicated malaria [36]. Though developed for clinical malaria therapeutic treatment, some of these drugs have been recommended for prophylaxis interventions as well. Sulfadoxine–pyrimethamine (SP), the most used drug combination for chemopreventive interventions, is recommended for (i) IPTp in malaria-endemic areas of Africa, (ii) IPTi for infants below 12 months of age in areas of moderate-to-high malaria transmission in Africa, and (iii), combined with amodiaquine, in monthly SMC for all children below 6 years during the transmission season [36]. Other drugs can also be used for prophylaxis purposes by travelers to endemic regions and residents in endemic areas.

Despite the interventions currently available against malaria, there are still limitations to reducing the burden of the disease. As an example, RDTs are fast, easy to perform and require neither electricity nor specific equipment, but advances in stability, affordability, detection of low parasitemia density, and identification of asymptomatic patients are needed to improve diagnosis and enable immediate treatment [31,39].

Much progress has been made in prevention strategies, mainly in mosquito vector control. The introduction of new guidelines such as IVM, a combination of vector control tools, has led to a reduction in transmission inside houses, thus decreasing the incidence of new infections and thereby the morbidity and the mortality of the disease [30]. Although these approaches have helped in eliminating malaria in certain regions [25,32], their impact on disease prevalence is usually limited in areas of high transmissibility. Developing novel outdoor vector control tools, adapting IVM strategies to the specificities of each region and health system, bringing new insecticides to the market, and developing efficient entomological surveillance systems are key points to improve prevention [25,30,31,32].

Although several antimalarial drugs for treatment and prophylaxis have been developed, the scenario is still far from optimal [40,41]. Factors such as costs (both for purchase and for continued drug supply), sustainability (a challenge for long-term programs), acceptability, poor product quality, and incorrect use leading to the evolution of resistance by *Plasmodium* are responsible for the decreasing efficacy of drugs [40,41,42,43]. In addition, the use of antimalarials for prophylaxis and chemoprevention purposes requires several doses and relies on adherence to the intervention, which may not happen, thus leading to resistance evolution. An effective drug specifically designed for prophylaxis objectives, for example, a single-dose drug, is needed for more efficacious interventions.

Despite the diagnosis tools developed recently, the prevention measures used in endemic countries, and the different antimalarial drugs available, an effective malaria vaccine remains a missing cornerstone to achieve the global goal of malaria elimination. Only one vaccine, RTS,S (Mosquirix), has completed phase III clinical trials, providing limited protection from severe malaria in African children [44]. In October 2021, Mosquirix was endorsed by the WHO for “broad use” in children, making it the first malaria vaccine to receive this recommendation. The development of a fully protective or transmission-blocking vaccine (or a combination of both) is imperative to achieve the goal of malaria elimination [23,24,31,32].

## 3. Nanotechnology against Malaria

The application of nanotechnology to health care has led to better knowledge of the biological mechanisms of diseases and to the development and improvement of tools for diagnosis and treatment [45]. Although the potential of nanomedicine against diseases of poverty and neglected diseases has not been fully exploited yet, new approaches and novel tools for malaria control, prevention, diagnosis, and treatment have been recently studied [46].

### 3.1. Diagnosis

Malaria diagnostic tools are mainly based on specific *Plasmodium* biomarkers, except for clinical diagnosis, which is based on the symptoms that a patient displays [47]. Biomarkers can be defined as indicators of the biological state of an organism, usually through the measurement of specific substances, processes, or structures from a removed sample [33]. These different biomarkers can be directly associated with the parasite density the patient is carrying, which can range from extremely low (below 1 parasite/µL) to high values (over 10,000 parasites/µL) [48]. Obtaining a fast and accurate result with a diagnostic test is essential for an appropriate treatment [48,49]. Nanotechnology has helped in identifying and fully characterizing malaria biomarkers as well as in developing novel sensors for diagnostic tests with the aim of improving quality, sensitivity, and reproducibility while diminishing the associated costs.

Currently there are six main *Plasmodium* biomarkers that serve as targets in diagnostic tests. These include five unique parasite proteins (histidine-rich protein II, lactate dehydrogenase, aldolase, glutamate dehydrogenase, and hypoxanthine–guanine phosphoribosyl transferase) and a pigment marker (hemozoin) [33]. They can be identified in a diagnostic test through a recognition element (e.g., antibodies or aptamers), producing a signal that is transduced into an output that can then be interpreted [33]. Apart from the three conventional malaria diagnosis tools (microscopic analysis, antibody-based RDTs, and NAATs), novel sensors have recently been developed. Aptamer-based sensors are of increasing interest because of aptamers’ good properties, such as high specificity towards the target molecule, stability, amenability to functionalization, and nonrequirement of animals for their production [50,51]. Different aptasensors have already been tested against *Plasmodium* biomarkers, placing them as simple, inexpensive, and rapid alternatives for malaria diagnosis [33,47,52,53]. Also attracting growing interest are electrochemical sensors, which are based on a recognition element that accounts for the selectivity of the specific biomarker, usually contained in a thin layer. Once the biomarker is detected, changes in electrical properties are produced (e.g., conductance or electric potential) and then transferred to the output [33]. This system has already been tested in electrochemical point-of-care devices and in label-free sensors [54], among others [33,53].

Nanotechnology can also be applied to improve diagnostic tools already in use. Immunosensors, which use antibodies for the detection of biomarkers, are the core element of most RDTs. Adding to these platforms nanomaterials such as gold NPs [55] and magnetic NPs [56], or designing multiplex immunoassays with the capacity to detect multiple biomarkers at the same time [57], can improve the sensitivity and the overall performance of sensors, resulting in a better and more precise diagnosis. Molecular methods for malaria diagnosis based on NAATs have also been improved through nanotechnology. Polymerase chain reaction was the first molecular diagnostic tool developed and it is currently widely used, although it has limitations in terms of cost, time, and skill requirements. Loop-mediated isothermal amplification has arisen as a simple, quick, specific, and cost-effective alternative allowing fast malaria diagnosis in remote areas [58,59,60]. Other devices are being developed, including microfluidic systems such as lab-on-a-chip sensors integrating multiple functions [61], although further research in this direction is needed.

### 3.2. Vaccines

Current malaria vaccine candidates can be divided into three different categories, depending on the stage of the *Plasmodium* cycle they target, which determines the vaccine’s purpose and how it works [62]. Pre-erythrocytic vaccines target the sporozoites travelling to the liver with the objective of inhibiting hepatocyte infection and the subsequent RBC invasion, thus preventing progression to symptomatic disease. Although the mechanism of this response has not been fully worked out yet, the response can be enhanced by triggering specific immune cells that target the circumsporozoite protein [63]. Most vaccine candidates targeting the pre-erythrocytic stage are in phase I and phase II clinical trials, except for the Mosquirix vaccine, which is the only malaria vaccine candidate having reached a pilot implementation phase III clinical trial [64,65]. It has an efficacy of around 45% in children aged 5–17 months and 30% in children 6-12 weeks old after four doses, and although its protection decreases over time, simulations confirm that it would be a good addition to other malaria prevention and control tools in order to decrease the incidence of severe malaria [62,66,67,68]. In April 2021, results of the phase IIb clinical trial of the vaccine candidate R21 adjuvanted with Matrix-M (R21/MM) were published [69]. This study assessed the efficacy of the vaccine in children aged 5-17 months from Burkina Faso. Participants were divided into three groups (receiving 25 µg R21/25 µg MM, 25 µg R21/50 µg MM, and the rabies vaccine in the case of the control group). All groups were administered three doses of the vaccine intramuscularly, and safety, immunogenicity, and efficacy were assessed over a 24-month period (endpoints after 6 and 12 months). The results showed a protection efficacy of 71% in group one and 76% in group two 12 months after the third dose [70]. This is the first malaria vaccine with an efficacy meeting WHO standards [71].

The second group, blood-stage vaccines, target infected erythrocytes with the generation of functional antibody response. Blocking parasite surface proteins and/or antigens on the membrane of pRBCs limits merozoite production as well as controlling and reducing parasitemia. As most of the acquired immune response of individuals with repeated episodes of malaria targets this stage, the development of an effective blood-stage vaccine is considered to be crucial to control the circulating parasites. Although there are different vaccine candidates targeting specific *Plasmodium* proteins exposed on pRBCs, none has shown efficacy against clinical malaria [66,68], probably because of the high polymorphism of the targeted antigens [62].

The third group comprises transmission-blocking vaccines (TBVs), which target gametocytes, the sexual parasite stages in the blood circulation. The aim of TBVs is to interrupt the transmission between humans and mosquitoes by causing an immune response to specific gametocyte proteins, reducing their infectivity. Although showing no individual benefit for the patients, TBVs are considered vital to reduce malaria prevalence in the population. Little is known of the efficacy of TBVs as all candidates are in phase I and phase II clinical trials [62,66,68].

**Figure 2:**
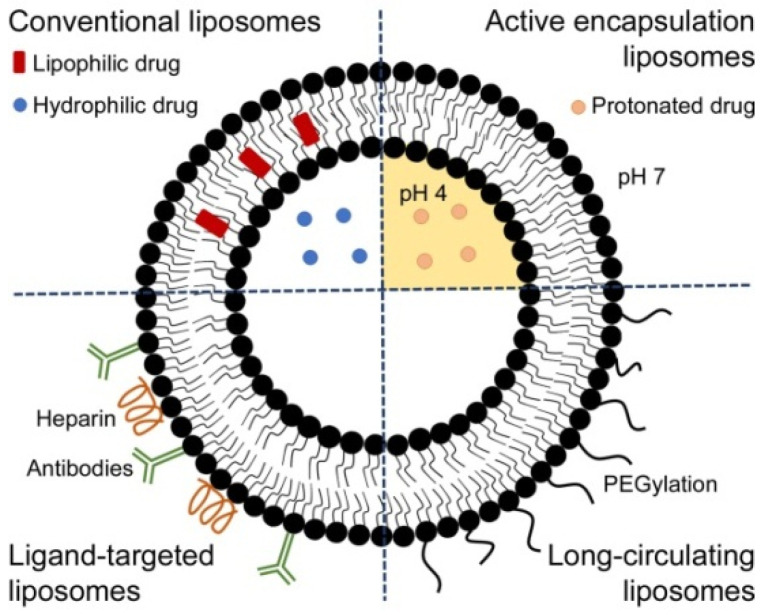
Schematic representation of liposomes formulated using different strategies aiming at improving the therapeutic efficacy of encapsulated drugs and the presentation of vaccine antigens. Conventional liposomes are formed by phospholipids and cholesterol and can encapsulate hydrophilic drugs in their aqueous cores and hydrophobic compounds in their phospholipid bilayers. Active encapsulating liposomes hold a pH gradient that can improve the loading of drugs with amphiphilic nature, which, depending on the pH, are found either in their protonated or deprotonated forms. Targeted liposomes are developed using specific pRBC ligands such as antibodies or heparin and promote the delivery of high drug doses with few side effects. Long-circulating liposomes can be formulated by modifying their surfaces with poly(ethylene) glycol (PEG) to enhance the blood residence time. Reproduced with permission from [14], The Royal Society of Chemistry, 2020.

Although much progress has been made towards a malaria vaccine, and several candidates have reached preclinical studies and are currently in the initial phases of clinical trials, only Mosquirix has completed phase III clinical studies [63]. To some extent, nanotechnology has played an important role in the development of malaria vaccines; as examples, Mosquirix is based on virus-like particles of nanometric dimensions targeting specific *P. falciparum* proteins [12,72], and vaccines based on lipid NCs are currently under study, as they may boost the immune response against malaria parasites [12,73,74,75,76]. Though the recent advances are encouraging, vaccines targeting different *Plasmodium* stages would be the ideal candidates; however, the production of such vaccines is more difficult. Several antigens can be combined in NCs in order to obtain wider and stronger immune protection. Liposomes are especially well suited to be functionalized with different ligands (Figure 2) and are actually widely used for vaccine production, as seen with coronavirus disease 2019 [77,78]. An integrated approach combining current malaria control strategies with an effective malaria vaccine would contribute to providing the long-term protection needed to advance towards malaria elimination [66].

### 3.3. Antiplasmodial Therapeutics

At present, there are several drug combinations for the treatment of clinical malaria, mainly ACTs. Although some of these drugs have shown good therapeutic efficacy, there is still room for improvement in avoiding side effects, reducing toxicity, increasing half-life, preventing drug resistance, and decreasing the dosage for effective treatment. Nanotechnology-based drug delivery systems are novel tools well placed to improve the efficacy of current antimalarial drugs and overcome their limitations. NCs can be designed to target specific molecules, protect drugs from degradation, prolong blood circulation time, cut down dose frequency, overcome side effects, improve pharmacokinetic (PK) profiles, and increase the overall efficacies of treatments [14,46,79,80]. To achieve these aims, there are three key NC components that need to be carefully assessed in the design phase: the drug loaded, the targeting molecules, and the core delivery system, each of which provides different qualities to the final NC formulation.

The first element that must be determined is the drug to be administered. In the case of malaria, the most active compounds currently available are ACTs, which are recommended by the WHO [36]. Although ACTs have been key in reducing global malaria incidence and deaths, with a huge impact in sub-Saharan Africa, the emergence and spread of resistance in the Greater Mekong region has been identified [81], posing the need to define new combination therapies, for example, triple ACTs [82]. Defining new combination therapies at the nanoscale is key when designing the NC formulation [17].

The second part that has to be defined are the targeting molecules, if any, to be added to the NC. Targeted delivery has the ability to increase local drug doses where the parasite resides, thus increasing treatment efficacy [17]. Considering the malaria parasite’s life cycle inside the human body and the available developed drugs, there are three different cell types that can be targeted: RBCs (and pRBCs), gametocytes, and hepatocytes [76]. Passive targeting can be achieved using conventional NCs for the accumulation of the active compound in the mononuclear phagocyte system [83], which may be useful for a slow release in blood, but not much for targeting RBCs. Another way to achieve passive targeting contemplates using surface-modified, long-lasting NCs, for example, with polymers such as poly(ethylene) glycol (PEG) [84], which increases the half-life in blood of the system [85]. This allows longer interactions with target cells and potentially less toxic side effects, as studied with halofantrine loaded in poly(lactic acid)-PEG NCs [86].

In contrast, active targeting can be achieved through the conjugation of specific ligands at the surface of the NC. These ligands allow a specific release of the drug to the desired cell or tissue [76], which is key in the delivery of antimalarial drugs to pRBCs. Urbán et al. demonstrated for the first time that, indeed, specific targeting could completely discriminate between pRBCs and RBCs [87]. Different molecules have been studied for this purpose, such as peptides, antibodies, heparin, and more recently DNA aptamers. Antibodies have been widely used [74,88,89], and interestingly, the natural polysaccharide heparin was also found to be a good pRBC-targeting molecule [90,91]. Finally, DNA aptamers are gaining momentum as targeting elements because of their highly specific binding to pRBCs, as seen in different studies [92,93]. Most of these ligands can be easily conjugated onto different NCs, allowing them to deliver the drug at the desired target sites.

Finally, the third element to consider is the actual capsule of the NC formulation, which protects and carries the drug. Liposomes (Figure 2) have the ability to transport hydrophilic and lipophilic agents and can be easily tuned in terms of size, surface charge, and functionalization. Liposomized drugs are protected from degradation, have increased plasma solubility and reduced toxicity and side effects, and can be targeted to specific sites to improve their PK profiles [14,79,80]. The application of liposomes in the delivery of antimalarial drugs has been widely studied. Liposomes encapsulating primaquine showed specific targeting and delivery to the liver and reduced toxicity and increased efficacy of the drug [73]; other nanoliposomes loading monensin also offered improved antiplasmodial activity [94]; and immunoliposomes encapsulating chloroquine and fosmidomycin reduced parasitemia by 20% and exhibited 10-fold increased efficacy over the free drugs [95]. More recently, nanoliposomes containing artemisinin and artemisinin derivatives have been studied, showing extended blood circulation, improved half-life time of the drugs, and lower toxicity [96,97]. Despite these good properties observed in different studies, liposomes present some limitations too: they are relatively expensive to formulate, are not free of potential toxic effects upon drug release following degradation, and are not adequate for oral administration, which is the preferred route in the vast majority of malaria cases [14,80].

Polymeric NCs can offer an alternative to liposomes as drug delivery vehicles, providing good solubility, reduced toxicity, biocompatibility, protection of the drug, specific targeting, and potential for different administration routes. Although they have limitations in terms of drug loading capacity, polymeric nanocarriers for antimalarial drugs have been widely studied [14,79,80,98,99]. Poly(lactic-co-glycolic acid) NPs conjugated with monensin showed 10-fold increased efficacy when compared to the drug alone [100] and exhibited a synergistic effect with controlled release for combination therapy of quinoline, chloroquine, and certain antibiotics [101]. Poly(amidoamine)-based NCs carrying chloroquine exhibited increased efficacy over the free drug due to the ability to selectively target pRBCs [102]. Polyaspartamide-based NPs increased the effectiveness of their conjugated drugs and were proposed as potential candidates to overcome drug resistance [103]. Different NPs carrying artemisinin derivatives also offered improved antiplasmodial efficacy in terms of sustained and controlled release, amelioration of water solubility, and blood circulation half-life of the drugs [104,105,106,107,108].

Other delivery systems such as micelles, nanocapsules, dendrimers, and hydrogels have also been studied as nanoformulations to improve the therapeutic outcomes of conventional antimalarial drugs. Not only do the previously discussed nanoformulations have the ability to increase the efficacy of the different drugs, but more importantly, they can contribute to overcoming drug resistance mechanisms evolved by *Plasmodium*. Still, global research in this direction is scarce, with few, if any, of the novel antimalarial nanoformulations developed being on a clinical trial pipeline [14,79,80]. A thorough revision of the available nanocarriers for antimalarial drugs has been recently published [14], and we therefore do not extend on this subject.

## 4. Next Generation of Antimalarial Medicines

Its potential for improving diagnostic tools, vaccines, the efficacy of conventional therapeutic drugs, and control and prevention strategies have placed nanotechnology as a cornerstone in malaria elimination. Sequencing the parasite genome allowed the identification of new targets in *Plasmodium*, and state-of-the-art technologies led to the discovery of novel active molecules against different stages of the parasite [109,110]. To ensure that the final products meet medical needs in terms of dosage, safety, efficacy, stability, and activity against resistant strains, a common description known as the Target Product Profile (TPP) was proposed [111]. TPPs may change according to external parameters and context, but they offer a framework for the minimally acceptable profiles of future medicines. As these will most certainly be a combination of different active agents to minimize the evolution of resistance by *Plasmodium*, a definition of the drugs entering clinical development has been proposed as well, known as the Target Candidate Profile (TCP) [111,112]. A crucial role in coordinating and advancing proposals for novel TCPs and TPPs enlarging the malaria drug discovery portfolio is played by Medicines for Malaria Venture (MMV) [113], which has updated its descriptions according to current needs (Table 1).

In the following sections, the ideal products for both treatment and prevention are outlined, highlighting the key points considering the target population to which they are addressed. Afterwards, the six more advanced next generation antimalarial candidates in the MMV pipeline [115] are discussed. For each of them, general information and status of the preclinical and clinical trials will be presented, and matching to the ideal product previously defined and possible limitations are assessed. Finally, the potential of nanomedicine to support the development of the ideal product is dealt with. To gather information and perspective for developing these sections, interviews and discussions with experts on malaria and nanomedicine were conducted (*transcribed in italicized text*).

### 4.1. Ideal Product Profile

Over the past years, the pipeline of drug development has seen a dramatic increase in terms of the number and diversity of molecules [114] with the discovery of new compounds having novel mechanisms of action [116]. This increase in potential antimalarial drugs, added to the global shift from malaria control to malaria eradication, stresses the need to define the ideal and minimally acceptable qualities of the new medicines as, according to experts, it is important “…*not to lose sight of the fact that you end up making a product that can have a lot of use but not the one you wanted.*” In that sense, two different TPPs were proposed, defined, and updated over the years: medicines for patient treatment (TPP1) and for chemoprevention (TPP2).

#### 4.1.1. TPP1: Case Management Medicines

The main objective of case management medicines is the treatment of acute, uncomplicated malaria in adults and children, as well as, ideally, severe malaria, although the main features may be slightly different in this second case. For that purpose, a combination of at least two molecules with demonstrated TCP1, TCP3, and TCP5 activity is desired, defining the ideal product as a single encounter radical cure and post-treatment prophylaxis (SERCaP) [117]. Such a medicine would be optimal, as “…*it will cure what you have, but what’s more, by having certain levels in blood for a certain period of time, the mosquitoes that bite you will no longer be able to cause the disease while the drug effects last*. *Once you develop the disease you need something highly effective that can kill the parasite, with what the WHO defines as 95% of efficacy for any new antimalarials*”. Indeed, the aim of a case management drug is to quickly cure malaria, because then, the patient stops suffering. Therefore, these molecules should have TCP1 activity, i.e., clearance of the asexual blood-stage parasitemia [114], the stage of the cycle in which the parasite replicates in the blood stream and the symptoms begin: “*The pre-erythrocytic phase is asymptomatic. When you have to treat people who are sick, they are in the erythrocytic phase, which is the one that you have to treat necessarily*”.

Apart from fast TCP1 activity, it is also essential to stop the transmission of malaria from the patient to the next mosquito and to limit the fast evolution of resistance by the parasite to the drugs: “*If you use only one drug, the parasite quickly develops resistance. Therefore, you need drugs with different mechanisms of action to reduce the emergence of resistance*”. This is one of the reasons why there is a need for a molecule with TCP5 activity, with the ability to block transmission by targeting gametocytes. Moreover, “*It would be ideal to have a drug that was also efficient against all human malarias and also against P. vivax hypnozoites. If we have a drug that can do it all, that would represent an important advantage*”. Indeed, that is the TCP3 activity demanded for all new drugs entering the MMV pipeline.

There is a general agreement that the best strategy to meet these requirements for new medicines is the combination of several different molecules, which, if possible, should be completely new compounds. The aim of combining two or more new molecules is to attack different stages of the *Plasmodium* life cycle without risking cross-resistance between them [114]. Following the identification of new molecules active against different *Plasmodium* stages, mainly with TCP1, TCP3, and TCP5 activity, the pipeline of medicines for the treatment of clinical malaria has been enlarged over the past years. Some of these new drugs are currently being tested in clinical trials in combination among themselves or with artemisinin derivatives [118].

Oral formulations will always be preferable; they should have a shelf life of at least 2 years, and the desired dosing regimen should be no longer than 3 days, ideally a single dose. Finally, the cost of the complete treatment course should not be higher than that of the current one, below 3 USD for adults and 1 USD for infants aged less than 2 years [114]. Other important aspects to consider are the PK profile of the drug and the dose needed to achieve the desired efficacy. Although new compounds are comparable to the current ACT treatment in these features, there are other mechanisms that can be explored to improve these properties, such as encapsulation in NCs and targeted drug delivery.

#### 4.1.2. TPP2: Chemoprotective Medicines

In addition to their therapeutic activity, antimalarial medicines can be also used as preventive treatment to reduce the incidence of malaria. TPP2 refers to both chemoprotection, the protection of subjects entering a high endemic area (for example, migrants or tourists), and chemoprevention, i.e., medicines administered to populations living in high endemic areas, e.g., for SMC [114]. In terms of chemoprotection, ideal antimalarial drugs should have liver stage activity (TCP4) and the possible benefit of long-lasting asexual blood stage activity (TCP1). Though TCP4 compounds are less prone to generating resistance, the presence of TCP1 molecules increases this risk. As these medicines are intended to be administered to large populations, safety is a major concern, and adverse effects should be avoided. Frequent administration of chemoprotective medicines is needed, and most drug candidates support only a once-weekly administration, although they should ideally support a once-monthly administration [114].

Medicines used for chemoprevention, for example, in intermittent strategies such as SMC, aim at maintaining high antimalarial levels in blood during the periods of increased malaria risk, usually the rainy season. SP combined with amodiaquine has been used for SMC campaigns [119,120,121], although resistance has been detected in several regions [122]. The WHO recommends using different medicines for malaria treatment and prevention and urges the development of novel drugs specific for prevention [36]. This idea was pointed out by some experts as well: “*Ideally you want to use for prevention drugs different from those that you are administering for treatment. There is a high risk of resistance evolving when prevention drugs are being used on a massive scale, e.g., on the whole population of newborns or through* *mass drug administration. Ideally, then, you do not want to compromise the drug that is being used as a first line of treatment for severe cases*”.

There are three key features that stand out for TPP2 medicines that have been identified in the literature and discussed during the interviews transcribed here. The first is related to safety: medicines aimed at chemoprevention and chemoprotection should have no drug-related severe adverse effects and minimal mild adverse effects [114]. Indeed, “…*a very important issue is that chemopreventive drugs have to be even safer than those used for treatment. If you are ill, you assume a certain risk. It is like the story of the COVID vaccines with AstraZeneca: when feeling bad one does not care if the medicine may have side effects, because in the end what you want it for is to save your life. On the other hand, when taking a drug for prevention, this drug must be much safer: you cannot accept the risks because you are healthy*”. In other words, the risk a patient is willing to assume is lower in a preventive treatment: “*It can be more difficult to convince a patient without any symptoms to take medicines*”.

The second key issue relates to the number of doses. Currently, recommended preventive treatments include three doses during a three-day course. Although theoretically, they present high clinical efficacy, the actual efficacy is much lower because of a lack of adherence to the whole regimen. Interviewed experts said that “*regarding prevention, it would be acceptable to lose a little bit of efficacy and have only one dose. The efficacy that you lose because people do not take the second and third doses is enormous. If there is a drug with a long half-life after a single dose, it would be advisable to sacrifice a little bit of efficacy in exchange for that benefit*”. The desired TPP2 medicines should have a PK profile allowing protection for at least one week, and ideally one month, with a single dose [114].

A third important parameter to consider is the administration route for the preventive medicines. The oral route is the preferred option, mainly because of acceptance by the population receiving it and the logistics for its administration: “*Where malaria is a real problem, and where you have up to 5–6 cases of malaria per child per year, one needs something oral that is easy to distribute, which does not need to be kept in the fridge, and which is as simple as possible. And oral is always easier than intravenous*”.

TPP2 medicines should have a minimal essential efficacy: they should offer at least an 80% reduction in the cumulative incidence of malaria, although this reduction should ideally be 95% or greater. It must be noted that the efficacy obtained in clinical trials may differ from the actual efficacy of the implementation because of different factors such as lack of adherence [36,123]. Overall, it is important for the prevention to be done intermittently, allowing patients to develop natural immunity against malaria: “*It is a balance: one needs to have the drug protecting people for as long as possible, but you cannot have the person permanently protected either. The development of natural immunity against malaria requires of the immune system to keep on fighting against infectious bites. Otherwise, the first episode of malaria after you stop being protected could be as serious as the first episode in a newborn or a traveler*”. The aim of preventive treatment is to reduce malaria incidence, but patients should develop immunity against malaria in order to attack and clear the parasite in case of contact.

Developing medicines with all the features discussed above is ambitious. Only a small number of molecules with potential TPP2 profile have entered the pipeline over the past years, although some of them have shown encouraging results [118].

### 4.2. Next Generation of Therapies Supported by MMV

**Figure 3:**
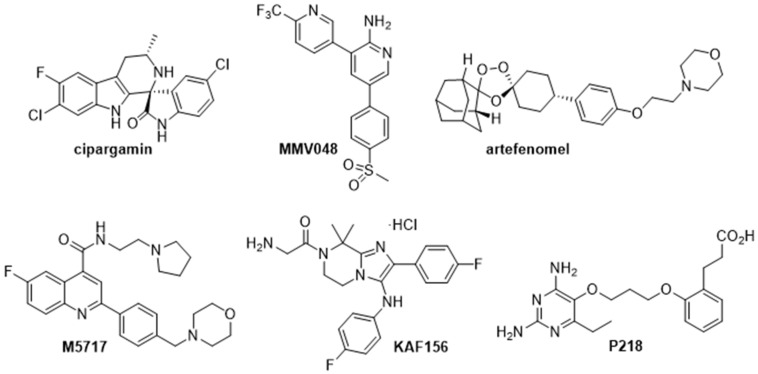
Chemical structures of the most advanced compounds in the current global antimalarial portfolio.

MMV has been supporting, over the last decade, projects for the discovery of new antimalarials, bringing to market a wide range of ACTs [115]. However, resistance to most of the ACT partner drugs has been detected recently, posing a major threat to malaria control and elimination and stressing the need to develop new molecules with novel mechanisms of action. MMV, together with its partners, is playing a major role in widening the pipeline of antimalarial candidates. Different drugs and combinations thereof are currently being tested in preclinical and clinical studies. Below, the most advanced compounds in clinical phases (Figure 3) are outlined and compared to the ideal products discussed in the previous section.

#### 4.2.1. KAF156/Lumefantrine

KAF156 (also known as ganaplacide) belongs to the imidazolopiperazine class, a novel antimalarial scaffold identified by a team form the Genomics Institute of Novartis Research Foundation [124]. It showed enhanced activity against the parasite and better biocompatibility compared to other formulations and can be synthesized through a simple, high-yielding, and scalable process [125]. The first preclinical studies indicated that KAF156 possessed antimalarial activity against different *Plasmodium* stages, suggesting that it could be used to prevent infection, treat acute malaria, and block transmission [126]. In phase I clinical trials, it presented a good PK profile, supporting a once-daily regimen for 3 days and even a single-dose regimen, both well tolerated and without adverse effects in humans [127,128]. In addition, KAF156 showed a rapid clearance rate against *P. falciparum* and *P. vivax*, even for strains resistant to current antimalarials [129]. At the moment, this drug is under phase IIb clinical trials in combination with a new formulation of lumefantrine, which presents improved bioavailability and allows a once-daily administration [130]. The objective of the study is to determine the most effective and tolerable lowest dosing of the combined drug [131]. Although early results are encouraging, in vitro studies have demonstrated the possibility that *Plasmodium* evolves resistance against KAF156/lumefantrine [132].

Overall, KAF156 presents several interesting properties placing it as a potential SERCaP candidate, with demonstrated TCP1, TCP3, and TCP5 as well as some TCP4 activity, the latter of which provides certain protection after treatment. Despite its multistage activity against *Plasmodium*, it showed slower parasitemia clearance when compared to ACTs. However, its potential to be used in single doses places it as a TPP1 case management drug. On the other hand, its known TCP4 activity also makes it a potential TPP2 prevention medicine, although the dose regimen in this case remains unclear. In conclusion, KAF156 has arisen as a candidate for the next generation of antimalarials, with improved properties compared to current ACTs except for the PK profile and parasitemia clearance time. More studies are needed to understand its mechanism of action and to further investigate efficacy and safety, as well as to fully characterize its activity when combined with lumefantrine.

#### 4.2.2. Artefenomel/Ferroquine

Artefenomel (also known as OZ439), the result of a large partnership coordinated by MMV [133,134], is a novel synthetic peroxide antimalarial candidate that shares some of the chemical groups of highly effective artemisinin derivatives [135]. It completed preclinical studies, showing good antimalarial properties and minimal adverse effects in rats [134]. Furthermore, it also showed interesting results in phase I clinical trials in terms of safety [136] and in phase II clinical trials in terms of parasitemia clearance after a single dose [137,138]. In addition, it exhibited initial activity against artemisinin-resistant *Plasmodium* strains [139]. However, it has some drawbacks, such as embryotoxicity when used during pregnancy and uncertainty about avoiding artemisinin-based resistance, as the data obtained in the studies were limited [135].

At the same time, ferroquine was developed as a derivative of chloroquine, but with the capacity to avoid the currently widespread *Plasmodium* resistance to the latter [139]. One outstanding property of ferroquine is its long half-life, placing it as a candidate for single-dose therapy against malaria when used in combination [118]. It also showed activity against ACT-resistant malaria [140]. It was first tested together with artesunate, but a novel artefenomel–ferroquine combination is currently in the MMV portfolio and under phase IIb clinical trials to determine safety and efficacy as a single-dose treatment [141]. This combination is a potential TPP1 candidate, presenting exceptional antimalarial properties in terms of TCP1 and TCP3 activity. Moreover, it has an interesting PK profile, with long half-life and rapid action against all asexual stages, and is a potential single-dose treatment, which is one of the key points of TPP1 medicines. However, there are some concerns, as it fails to attack all gametocytes, showing only modest TCP5 activity.

#### 4.2.3. Cipargamin

Cipargamin, formerly known as KAE609, an antimalarial candidate belonging to the spiroindolone family, was the outcome of a partnership among Novartis, MMV, Wellcome Trust, and the Swiss Tropical and Public Health Institute [113]. This distinct class of compounds has a novel mechanism of action against all parasitic blood stages [142]. Cipargamin was identified after screening and lead optimization of thousands of compounds [143], having neither adverse effects nor significant cytotoxicity in in vitro and in vivo preliminary studies. It demonstrated activity against *P. falciparum*, attacking different stages of the parasite [144], including gametocytes, placing it as a transmission-blocking antimalarial supporting single- or multiple-dose regimens [145].

Phase I clinical trials to assess cipargamin’s PK profile (aimed at determining the effective dose causing 100% reduction in parasitemia) and safety and tolerability assays in humans were conducted, with the main concerns being hepatotoxicity and mild side effects [142,146,147]. Interestingly, in a phase II clinical study conducted in Thailand, cipargamin showed rapid efficacy in the treatment of *P. falciparum* and *P. vivax* malaria [147]. Currently, the first in-human study with an intravenous formulation of cipargamin has been completed, and a phase II study in severe malaria patients is underway [148].

Although promising in preclinical and clinical studies, cipargamin has some limitations. More studies to find a suitable companion drug for an effective combination therapy should be undertaken, since combination therapies have proven to be more effective in the prevention of resistance than single-drug therapies. Indeed, in vitro resistance to cipargamin was found in some studies, although other antimalarial drugs were effective against those resistant strains [144]. Because of its novel mechanism of action, cipargamin exhibited fast TCP1 activity, even against known resistant strains, plus TCP5 activity, showing its potential as transmission-blocking drug. Moreover, its PK profile could support a single-dose treatment. It therefore presents itself as an interesting TPP1 candidate. Nevertheless, it presents some concerns in terms of safety and lacks a suitable combination partner to date. Such a partner should present TCP3 activity to fully comply with TPP1 medicinal features.

#### 4.2.4. MMV048

MMV048, known also as MMV390048, resulted from a collaboration between the Griffith Research Institute for Drug Discovery and a team from the University of Cape Town [124]. It is a novel antimalarial compound from the aminopyridine class that has entered phase I clinical studies in Africa [149]. MMV048 demonstrated activity against different asexual blood stages of the parasite, with its peak of efficacy against the schizont form. In addition, it was active against gametocytes, placing it as a multistage antimalarial candidate suitable for chemoprevention, treatment, and transmission-blocking interventions [124]. Preclinical analysis in animals confirmed its potential as a single-dose curative therapy, as a transmission-blocking drug, and as a prophylactic agent because of its long half-life when administered orally and its activity on liver stages, although its effect against late-stage hypnozoites remains unclear [150]. Three different phase I in-human studies were performed to assess the safety, tolerability, and PK profile of MMV048, with results suggesting that it was well tolerated, with the potential to be used as a preventive medicine and even as a single-dose case-management drug [151,152,153,154]. Currently, a phase IIa clinical trial to confirm its observed activity in malaria patients is being conducted in Ethiopia [123,155]. Despite good results in preclinical and early clinical studies, concerns regarding the precise safe dosage and potential combination drug partners remain key points for the future development of MMV048. Its teratogenicity has recently led to its discontinuation by MMV [156].

#### 4.2.5. M5717

M5717, formerly known as DDD498, is the product of a collaboration between MMV and the Drug Discovery Unit from the University of Dundee. M5717 belongs to the family of quinoline-4-carboxamide scaffolds and acts by inhibiting *P. falciparum* elongation factor 2, a novel mechanism of action among antimalarials [123,157]. In preclinical studies, M5717 showed excellent blood stage activity without any clinically relevant safety concern, confirming its potential for single-dose treatment and once-weekly chemoprotection [158]. A phase I clinical trial to assess its safety, tolerability, PK profile, and parasite clearance in healthy subjects following infection with *P. falciparum* was conducted (results not posted yet) [159], and another phase I clinical trial to assess the preventive activity of a single oral dose is currently under way [160]. The results of these studies will hopefully confirm antimalarial activity in human subjects. With its novel mechanism of action, M5717 showed good TCP1 and TCP5 activity, presenting it as a TPP1 candidate. Moreover, its long half-life may allow for a single-dose regimen when combined with a fast-acting molecule. Finally, it can also be considered as a TPP2 candidate because of its activity against liver-stage schizonts. More studies testing its efficacy, safety, and PK profile are needed, as well as an assessment of the likelihood of future resistance evolution and of potential combination partners.

#### 4.2.6. P218

P218 is being developed through a collaboration between MMV and Janssen; it is a long-acting, single-dose injectable drug for prevention [115]. It showed activity against blood stages of *Plasmodium*, but it raised much interest as a chemopreventive agent because of its outstanding activity against *P. falciparum* schizonts in the liver stage [161]. A first-in-human phase I clinical trial was conducted, concluding that it had a favorable safety, tolerability, and PK profile, thus confirming its potential for malaria chemoprotection [162,163]. A second phase I clinical trial was also performed, with results confirming its safety and tolerability with excellent protective efficacy [161,164]. Despite these encouraging perspectives, the short half-life of P218 may be a barrier for its future development as a chemoprotective agent [161,162], which has led to this drug being discontinued by MMV [156].

## 5. Nanotechnology Applied to the Next Generation of Antimalarials

Nanomedicine has had a great impact in health care, leading to the development and improvement of tools for disease diagnostics and treatment. In malaria, nanomedicine has been applied mostly in the development of novel therapeutics, in which the active compounds are conventional drugs already in use combined with a delivery system [165]. This has allowed the optimization of certain features of the drug, such as its PK profile or efficacy. Moreover, in certain cases, the new medicine may overcome the drug resistance mechanisms evolved by *Plasmodium*. Nanotechnology could play a key role in the development of the next generation of antimalarial medicines, improving the features of existing and future drugs in order to meet the specific and desired TPP1 and TPP2 criteria. Interviewed experts said that “*nanomedicine can provide different structures, which by being different from the encapsulated drug and having a different biological behavior, can be considered almost new drugs. However, the active principle, the mechanism of action that you transport, is that of the original drug*”. As a clear example, nanotechnology could be instrumental in bringing back to the clinical pipeline drugs that have been discontinued because of high toxicity or a short half-life, such as MMV048 and P218 [156].

**Figure 4:**
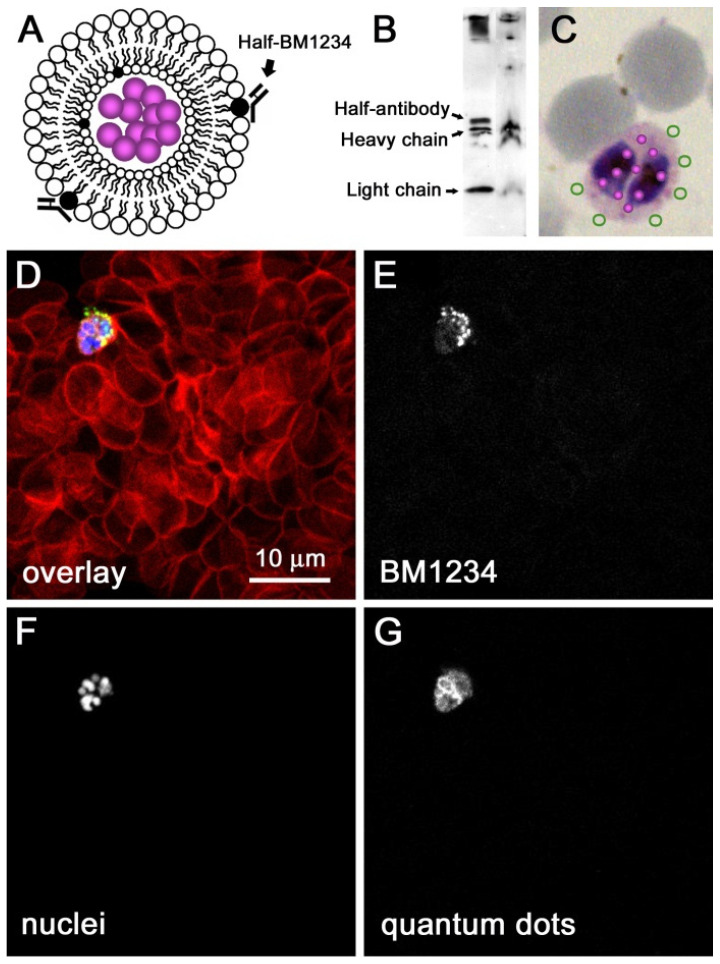
Confocal fluorescence microscopy analysis of the delivery of immunoliposome cargo to pRBCs. (**A**) Cartoon showing a quantum dot-containing liposome functionalized with half-antibodies. (**B**) Western blot analysis of the conjugation to liposomes (right lane) of the pRBC-targeting BM1234 antibody. Immunoliposomes were purified by ultracentrifugation prior to electrophoresis. (**C**) Graphical scheme of the expected performance of nanovectors when added to a *P. falciparum* culture containing both infected and noninfected cells. (**D**) Confocal fluorescence microscopy section of a suspension of RBCs containing ca. 5% pRBCs that had been treated, for 90 min and prior to fixation, with a preparation of immunoliposomes assembled as depicted in (**A**). The selected field contained a single pRBC among tens of noninfected cells, showing the fluorescence of RBC plasma membranes (red), antibody detection (green), quantum dots (white), and nuclei (blue). For easier visualization of the colocalization of quantum dots and antibodies only in pRBCs, the fluorescence signals for (**E**) antibodies, (**F**) nuclei, and (**G**) quantum dots are shown separately in white. Reproduced with permission from [87], Elsevier B. V., 2011.

One of the properties on which the delivery system may have a greatest impact is safety. When combining a drug with a specific NC, undesired interactions can be avoided, and thus, the toxicity of the drug can be lowered. Moreover, specific targeting provides large local concentrations of the drug near the parasite while keeping a low overall concentration in the organism. Although most of the newly discovered drugs present good safety profiles, adding NCs to the formulation makes them even safer. Another property that can be modulated by incorporating NCs is the PK profile: with specific targeting and controlled release, attractive PK profiles can be achieved that are adapted to the needs of TPP1 and TPP2. Drug-containing liposomes specifically targeted to pRBCs (Figure 4) can be a good choice for the encapsulation of antimalarials for intravenous administration in severe malaria cases [87,95,166,167,168].

**Figure 5:**
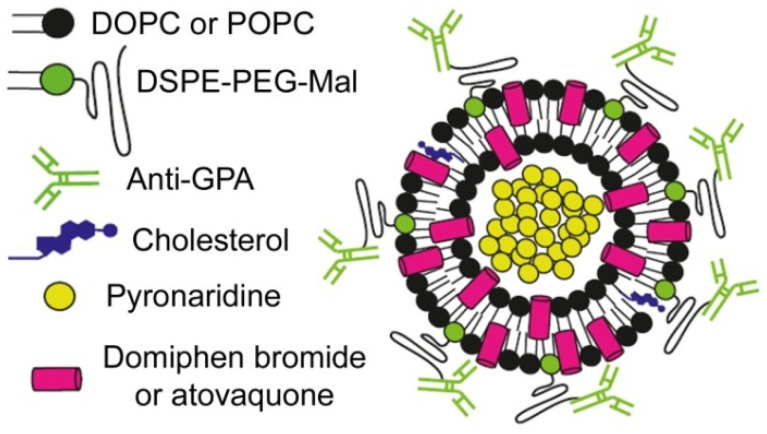
Scheme of an immunoliposome designed for targeted antimalarial combination therapy. DOPC: 1,2-dioleoyl-*sn*-glycero-3-phosphocholine; POPC: 1-palmitoyl-2-oleoyl-*sn*-glycero-3-phosphocholine; DSPE: 1,2-distearoyl-*sn*-glycero-3-phosphorylethanolamine; Mal: maleimide; GPA: glycophorin A. Reproduced with permission from [172], MDPI, 2019.

Some of the new antimalarials in the clinical pipeline described above have protonatable groups that can be used for their encapsulation in the liposomal lumen through pH gradient strategies, providing highly efficient drug loading [169,170,171]. Other compounds with stronger lipophilic characters are amenable to incorporation into the liposome lipid bilayer, allowing for the engineering of liposomal nanocarriers encapsulating two or more drugs, which can boost the prospects of combination therapies (Figure 5).

**Figure 6:**
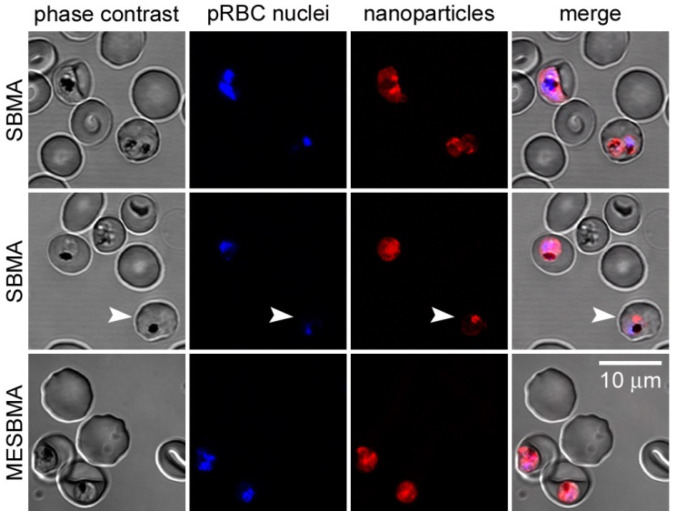
Confocal fluorescence microscopy analysis of the interaction of curcumin-containing, Cy3-labeled PBMA-SBMA and PBMA-MESBMA nanoparticles with RBCs and pRBCs. The arrowhead indicates an early trophozoite-stage parasite. Reproduced with permission from [173], Elsevier B. V., 2021.

Achieving oral formulations with NCs is key to developing new TPP1 and TPP2 medicines: “*The main problem (a drug) will face is to be able to withstand the passage through the stomach. There it will find a very acidic and destructive environment. Furthermore, it should be absorbed by the intestinal system. There are oral formulations made with certain types of polysaccharides, or structures that resist passage through the stomach and allow a certain degree of intestinal absorption*”. Some zwitterionic nanoparticles based on poly(butyl methacrylate-co-morpholinoethyl sulfobetaine methacrylate) (PBMA-MESBMA) targeting pRBCs (Figure 6) have shown good activity in encapsulating certain antimalarial compounds, such as curcumin, helping them reach the blood circulation following oral administration [173]. Other polymers based on dendrimeric structures with the capacity for drug encapsulation have also provided good pRBC targeting [174,175]. Nanotechnology could also be applied in other administration routes, such as sprays containing the medicine, which can be internalized through the lungs, or via subcutaneous injection or transdermic pads, allowing slow release of the drug, which could be an alternative in certain cases.

**Figure 7:**
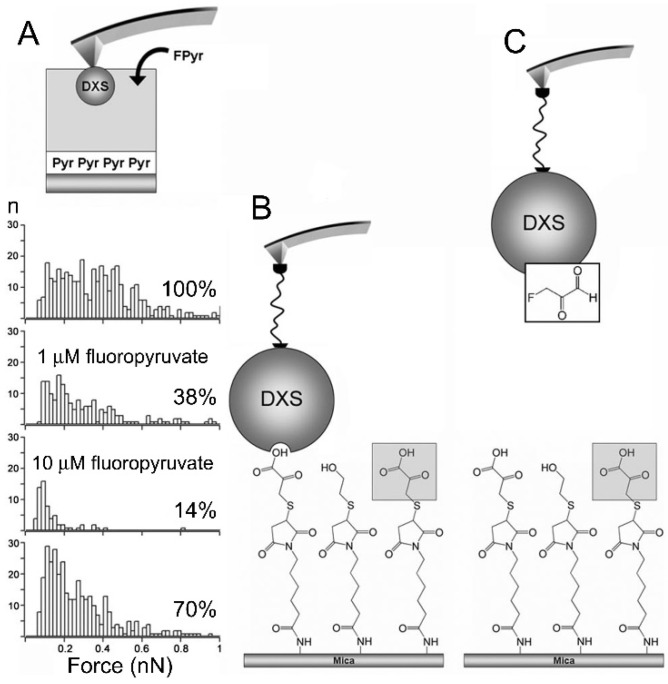
High-sensitivity, single-molecule nanosensor for the detection of DXS inhibitors. (**A**) Single-molecule force spectroscopy analysis of the effect of soluble fluoropyruvate on the binding between immobilized DXS and pyruvate. Temporal sequence from top to bottom: no soluble fluoropyruvate, addition of 1 μM fluoropyruvate, addition of 10 μM fluoropyruvate, removal of soluble fluoropyruvate. (**B**) Configuration showing the interaction between DXS bound to a nanoscale sensor and pyruvate to mica surfaces. (**C**) A significant decrease in the binding of DXS to the pyruvate-functionalized surface indicated the presence of an inhibitor in solution. Reproduced with permission from [176], Federation of American Societies for Experimental Biology, 2010.

Because the key component of NCs is the drug to be administered, it is essential that new candidates enter the pipeline of medicines against malaria, as these will be the active molecules of future delivery systems. It is necessary to fully characterize the mechanism of action of new drugs, e.g., defining which stages of the *Plasmodium* life cycle they interfere with, the interactions they have with the parasite, and other properties such as their PK profiles. Nanotechnology can also offer solutions for the discovery of new antimalarial drugs, for instance, with the development of highly sensitive, single-molecule methods for the identification of inhibitors of enzymes essential for malaria parasites. Using single-molecule force spectroscopy (SMFS), the interaction between the first enzyme of the 2-C-methyl-D-erythritol-4-phosphate pathway essential for the viability of the malaria parasite, 1-deoxy-D-xylulose 5-phosphate synthase (DXS), and its two substrates, pyruvate and glyceraldehyde-3-phosphate, was characterized (Figure 7) [176]. The DXS inhibitor fluoropyruvate was detected by such SMFS nanobiosensors at a concentration of 10 μM, which improved by two orders of magnitude the sensitivity of conventional enzyme activity assays. This result highlights the potential of individual enzyme–substrate handling for the biodiscovery of new antimalarial and antibiotic compounds present in natural product extracts at concentrations well below the detection limits of current enzymatic assays.

The impact that nanoscience can have on the development of the next generation of antimalarial treatments could be large. The main concern about nanotechnological solutions for malaria lies in the costs of the research itself and the final medicine production. Big pharma is not attracted to invest in pathologies such as malaria, as it may be less lucrative than investing in other type of diseases more prevalent in high-income regions, such as cancer or neurological disorders. However, nanotechnology can offer cost-affordable products with curative doses close to the WHO recommendations for endemic countries. To bypass the need for clinical trials, antimalarial drug nanocarriers active against the mosquito stages of *Plasmodium* can be designed to be directly delivered to *Anopheles* [177,178,179]. Eliminating malaria parasites through the delivery of targeted nanocarriers directly to the mosquito is the objective of the EuroNanoMed project NANOpheles [180], the final results of which will provide some interesting nanoformulations capable of blocking the sexual part of the pathogen’s life cycle, which occurs in the insect vector. Although delivering drugs to mosquitoes presents some obvious challenges, the potential gains of such strategy are evident in terms of the economic landscape of malaria.

## 6. Conclusions

Despite the recent approval for widespread use of the first malaria vaccine, its moderate efficacy (30%) and high price (5 USD per dose) [181] call for continued research efforts. Nanotechnology and nanomedicine can offer strategies to develop a successful toolkit for the fight against malaria, both by improving conventional actions and through developing new products. This could make the global goal of malaria control and elimination, and further eradication, feasible. However, to achieve this objective, significantly more investment by public institutions and industrial partners is needed in this field. The vast majority of nanomedical interventions have been focused on noncommunicable diseases; it is time to fully translate this knowledge into research against diseases of poverty and neglected diseases, which will stimulate the development of innovative approaches to diagnose, prevent, and treat them. Finally, the transfer of this knowledge to low- and mid-income countries is crucial to allow them becoming active agents in the invention and uses of these new tools.

## Figures and Tables

**Table 1:** Updated description of TPPs and TCPs. Adapted from [114], Springer Nature, 2017.

Profile	Use
TPP1	Case management medicines
TPP2	Chemoprotection medicines
TCP1	Molecules that clear asexual blood-stage parasitemia
TCP3	Molecules with activity against hypnozoites
TCP4	Molecules with activity against hepatic schizonts
TCP5	Molecules that block transmission by targeting parasite gametocytes
TCP6	Molecules that block transmission by targeting the insect vector
